# P-746. Characterizing Deep Neck Infections (DNI) at a Tertiary Care Center in Philadelphia with a High Population of People Who Inject Drugs (PWID)

**DOI:** 10.1093/ofid/ofae631.942

**Published:** 2025-01-29

**Authors:** Kaya Patel, Stephanie Spivack, Flora Yan, Noah Thorntan, Karl Whitley, Ahmed Soliman, Sara K Schultz

**Affiliations:** Perelman School of Medicine , Philadelphia, Pennsylvania; Temple University Health System, Philadelphia, Pennsylvania; Temple University Hospital, Philadelphi, Pennsylvania; Temple University Hospital, Philadelphi, Pennsylvania; Temple University Hospital, Philadelphi, Pennsylvania; Lewis Katz School of Medicine, Philadelphia, Pennsylvania; Temple University Hospital, Philadelphi, Pennsylvania

## Abstract

**Background:**

Acute bacterial skin and skin structure infections (ABSSSI) including cellulitis and abscess are common complications of intravenous drug use (IVDU). With the widespread introduction of xylazine in the Philadelphia drug supply since 2019, more patients are injecting into their neck due to limited peripheral access from vein sclerosis or xylazine wounds. Herein, we have characterized DNI in PWID and their outcomes.

Figure 1

**Figure:**
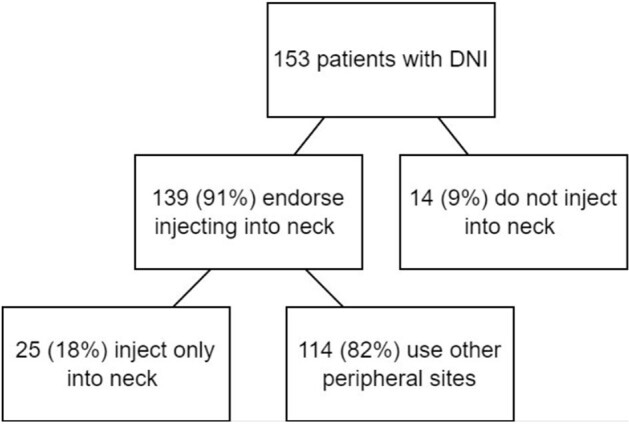

Injection behavior

**Methods:**

The IRB approved this study. We conducted a retrospective chart review of patients with documented opioid use disorder (OUD) with injection behavior and a diagnosis of DNI from January 2016 through December 2022. We collected data on demographics, microbiology, drug use and treatment to characterize infection, surgical intervention, and outcomes.

**Figure 2.**
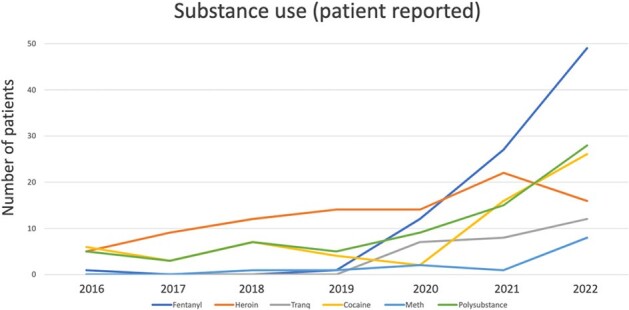
Substances used

**Results:**

We identified 153 patients with OUD and injection behavior who were diagnosed with DNI. 80 (52%) patients were male and 73 (48%) were female. 101 (66%) were experiencing homelessness. 139 (91%) patients reported injecting into their neck and 25 (16%) patients reported using their neck as the only site of injection due to limited peripheral access. Figure 1. 83 (54%) endorsed polysubstance drug use with cocaine or amphetamines. Figure 2. Types of infection include: 131 (86%) neck abscess, 22 (14%) neck phlegmon, 14 (9%) internal jugular (IJ) septic thrombophlebitis, 3 (2%) sternoclavicular septic arthritis, 3 (2%) with mediastinal involvement, and 2 (1%) osteomyelitis (OM) of clavicle or mandible.

124 (81%) patients underwent drainage. 103 (83%) of these patients had cultures sent from the abscess. The predominant organisms were MRSA (47, 46%), MSSA (15, 15%), GAS (23, 22%), and alpha-hemolytic Strep (26, 25%). Figure 3. 137 (90%) patients had blood cultures sent, 115 (84%) without growth, 10 (7%) MRSA, 10 (7%) GAS, and 3 (2%) MSSA. 21 (17%) required repeat intervention. Within 30 days there were 48 (31%) readmissions and 1 (0.5%) death.

**Figure 3.**
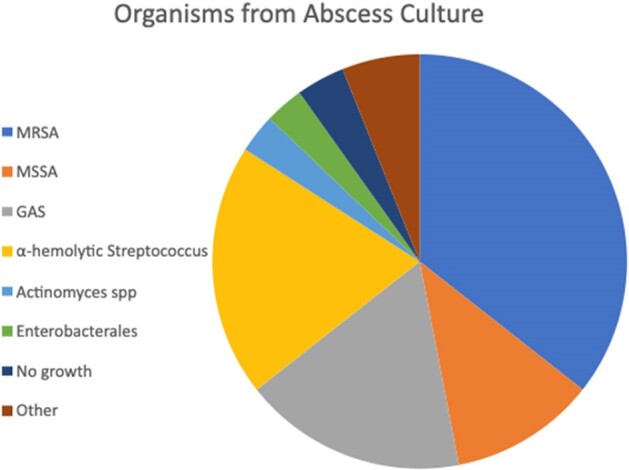
Microbiologic data from abscess cultures

**Conclusion:**

Since 2016, there has been a sixfold increase in incidence of DNI in PWID. Figure 4. The complications of neck infection can be catastrophic with extension into mediastinum, septic thrombophlebitis, bloodstream infections, or bone and joint infections. It is important to recognize neck infections early and intervene as soon as possible before further complications occur.

**Figure 4.**
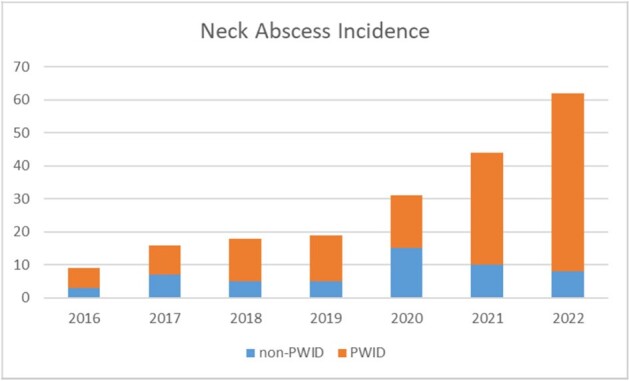
Neck abscess incidence

**Disclosures:**

**Sara K. Schultz, MD FACP FIDSA**, AbbVie: Advisor/Consultant

